# Surface Modification of Curcumin Microemulsions by Coupling of KLVFF Peptide: A Prototype for Targeted Bifunctional Microemulsions

**DOI:** 10.3390/polym14030443

**Published:** 2022-01-22

**Authors:** Rungsinee Phongpradist, Wisanu Thongchai, Kriangkrai Thongkorn, Suree Lekawanvijit, Chuda Chittasupho

**Affiliations:** 1 Department of Pharmaceutical Sciences, Faculty of Pharmacy, Chiang Mai University, Chiang Mai 50200, Thailand; rungsinee.p@cmu.ac.th; 2 Center of Excellence for Innovation in Analytical Science and Technology for Biodiversity-Based Economic and Society (I-ANALY-S-T_B.BES-CMU), Chiang Mai University, Chiang Mai 50200, Thailand; 3 Chemistry Program, Faculty of Science and Technology, Pibulsongkram Rajabhat University, Phitsanuloke 65000, Thailand; wisanuthongchai@psru.ac.th; 4 Department of Companion Animals and Wildlife Clinic, Faculty of Veterinary Medicine, Chiang Mai University, Chiang Mai 50100, Thailand; kriangkrai.th@cmu.ac.th; 5 Department of Pathology, Faculty of Medicine, Chiang Mai University, Chiang Mai 50200, Thailand; suree.lek@cmu.ac.th

**Keywords:** microemulsions, modified Pluronic, targeted, surface modification, KLVFF, curcumin

## Abstract

Curcumin is one of the most promising natural therapeutics for use against Alzheimer’s disease. The major limitations of curcumin are its low oral bioavailability and difficulty in permeating the blood–brain barrier. Therefore, designing a delivery system of curcumin to overcome its limitations must be employed. KLVFF, a peptide known as an amyloid blocker, was used in this study as a targeting moiety to develop a targeted drug delivery system. A prototype of transnasal KLVFF conjugated microemulsions containing curcumin (KLVFF-Cur-ME) for the nose-to-brain delivery was fabricated. The KLVFF-Cur-ME was developed by a titration method. A conjugation of KLVFF was performed through a carbodiimide reaction, and the conjugation efficiency was confirmed by FTIR and DSC technique. KLVFD-Cur-ME was characterized for the drug content, globule size, zeta potential, and pH. A transparent and homogeneous KLVFF-Cur-ME is achieved with a drug content of 80.25% and a globule size of 76.1 ± 2.5 nm. The pH of KLVFF-Cur-ME is 5.33 ± 0.02, indicating non-irritation to nasal tissues. KLVFD-Cur-ME does not show nasal ciliotoxicity. An ex vivo diffusion study revealed that KLVFF-Cur-ME partitions the porcine nasal mucosa through diffusion, following the Higuchi model. This investigation demonstrates the successful synthesis of a bifunctional KLVFF-Cur-ME as a novel prototype to deliver anti-A*β* aggregation via an intranasal administration.

## 1. Introduction

Alzheimer’s disease (AD) contributes to 60–70% of dementia cases, mostly found in the elderly [1]. At present, the pathophysiology of Alzheimer’s disease is still not clear, but the beta-amyloid plaque (A*β*) is one of the pathological hallmarks, and is considered a target for treatment. Current therapeutic agents, which are available to treat AD, fall into two categories, including drugs that only treat cognitive symptoms and drugs that may delay clinical decline by removing A*β* [2]. Most therapeutic agents are in the form of tablets and capsules, which cause issues for patients in late-stage AD with swallowing difficulties [3]. These reasons led to the development of various drug formulations administered through routes other than oral. The delivery of the drugs directly to the brain has been shown to improve the efficacy of the treatment [4]. Nowadays, the intranasal administration (IN) of drugs has gained attention because of several advantages, including the direct access of drugs from the nasal cavity to the central nervous system, non-invasive administration, convenience for self-medication, and improved patient compliance [5,6]. In addition, intranasal to brain delivery can also avoid the blood–brain barrier and hepatic firss-pass metabolism, which are the main obstacles of oral drug delivery [7,8]. Notwithstanding, intranasal drug delivery encounters the restriction of a poor drug diffusion through the nasal mucosa, leading to an insufficient concentration of drug delivery to the desired target site and, thus, the therapeutic effect cannot be achieved [6].

Nanosize drug delivery systems provide an improved delivery from the nose to the brain because they can enhance a drug’s solubility, permeability, and help protect the encapsulated drugs from chemical and physical degradation [8,9,10]. In addition, nanoscale drug delivery systems, particularly through passive targeting, have been hypothesized to allow the drugs to reach the target tissue and release the active drugs [11]. Over past decades, microemulsions (ME) has also attracted much interest as a drug delivery system and it has been reported as a suitable carrier of drugs for solving the solubility problem [5,12]. ME consists of an aqueous phase, an oil phase, and a mixture of a surfactant and co-surfactant [13]. In general, ME is a transparent, optically isotropic, and thermodynamically stable system [14]. ME provides definite advantages, including thermodynamic stability, a high solubilizing capacity for the lipophilic drugs, enhancing permeability through the biological membranes, increasing bioavailability, and reducing the inter-and intra-individual variability in drug pharmacokinetics [5,12]. Numerous studies have shown that microemulsions can be effectively applied as drug carriers for several routes of administration, including intranasal administration [5,13,15,16,17]. Microemulsions of carbamazepine and rivastigmine have shown to increase the intranasal uptake of drugs into the brain [5,15,18].

The transportation of drugs directly to the targeted pathophysiology of the disease is the goal of the treatment, because targeted therapy raises the therapeutic effects while minimizing undesired effects of drugs on the untargeted sites. Surface-modified nanocarriers can be delivered to the target site, such as the CNS, through a conjugation of the targeting moiety, which is the ligand for specific proteins that specifically present in the pathophysiology of the disease [9,19]. Many researchers have investigated the targeting delivery for CNS treatment, including the surface modification of the nanocarrier [20,21,22,23,24]. However, to our knowledge, surface-modified microemulsions for nasal-to-brain targeting drug delivery have not been reported previously; hence, the development of this system would provide a promising novel strategy for intranasal administration. One of the pathological hallmarks of AD is A*β* aggregation [25]. Thus, a molecular modulator that is specific to A*β* aggregation could be used as a targeting moiety to prevent and treat AD, specifically. Interestingly, the KLVFF peptide was reported as an inhibitor of A*β* aggregation [26,27]. KLVFF was first synthesized by Tjernberg et al. [28]. It is a peptide sequence containing 16–20 residues, designed from the full-length peptide, which hinders A*β* fibrillogenesis, resulting in the inhibition of amyloid aggregation [28,29]. KLVFF contains a diphenylalanine sequence, which plays a significant role in amyloid aggregation by forming highly extended fibrils with a *β*-sheet structure [30]. KLVFF could bind the confined region of the A*β* strand and interfere with the attachment of neighboring A*β* strands for assembly and aggregation [27]. Thereafter, KLVFF was grafted with drug carriers for A*β* plague targeting [31]. 

Curcumin, a phenolic compound, is a low-molecular-weight molecule derived from the rhizome of *Curcuma longa* L., a member of the Zingiberaceae family [32,33,34]. Several pharmacological activities of curcumin have been reviewed, including its antioxidant, antimicrobial, antitumor, and anti-inflammation capabilities [32,33]. Strong evidence indicated a neuroprotective action of curcumin against AD [34,35]. Curcumin demonstrated the strongest inhibitory effect in preventing fibril formation among 214 antioxidant compounds [36]. It is known as a potent inhibitor of A*β* aggregation [37]. Curcumin has been reported to reduce the *β*-sheet content of the peptide, destabilize and disaggregate A*β* fibrils, decrease the toxicity of A*β* oligomers, and reduce amyloid formation in vivo [34,37,38]. However, the obstacles of curcumin being used for clinical applications are its low hydrophilicity, rapid metabolization, poor absorption, low bioavailability, low permeability, and lack of targeting capabilities [34,39]. Hence, drug delivery systems are addressed to overcome such drawbacks to improve the biological and pharmacological activity of curcumin. 

The strategy for this study is to combine the merits of targeted drug delivery systems with the transnasal route of administration. We propose that binary inhibitors (curcumin and KLVFF) of amyloid aggregation, which have different binding sites, would additively or synergistically inhibit A*β* aggregation. This study describes the synthesis and characterization of surface-modified microemulsions encapsulating curcumin. In addition, the microemulsions containing curcumin are coupled with the KLVFF peptide for specific binding to A*β* fibrils. A bifunctional microemulsions was hypothesized to have dual modes of action, i.e., the A*β* aggregation inhibition of curcumin and KLVFF, and the targeting delivery property of the KLVFF peptide. The physicochemical properties, a chemical analysis, and nasal ciliotoxicity study were investigated to confirm the possibility of curcumin microemulsions conjugated using the KLVFF peptide as a prototype for bifunctional microemulsions for the treatment of AD via the intranasal route. 

## 2. Materials and Methods

### 2.1. Experimental Materials

Pluronic F-127^®^ was a gift from BASF chemical company (St. Louis, MO, USA). 1-Ethyl-3-[3-dimethylaminopropyl]-carbodiimide hydrochloride (EDC), N-hydroxysulfosuccinimide (sulfo-NHS), tetrahydrofuran (THF), dimethylaminopyridine (DMAP) succinic anhydride polysorbate 80 (TWEEN 80), Sorbitan monolaurate (Span 20), glycerine, propylene glycol, polyethylene glycol 400 (PEG 400), and isopropyl myristate (IPM) were purchased from Sigma-Aldrich (St. Louis, MO, USA). Ethanol, methanol, and carbon tetrachloride were obtained from Sigma-Aldrich (Steinheim, Germany). Curcumin was obtained from Merck (Darmstadt, Germany). KLVFF peptide was synthesized and purified by PEPMIC (Suzhou, China). Monobasic potassium phosphate was purchased from RCI Labscan (Bangkok, Thailand). Sodium hydroxide was obtained from KEMAUS (New South Wales, Australia).

### 2.2. Synthesis of Carboxylated Pluronic (COOH-Pluronic F127)

**Figure 1:**
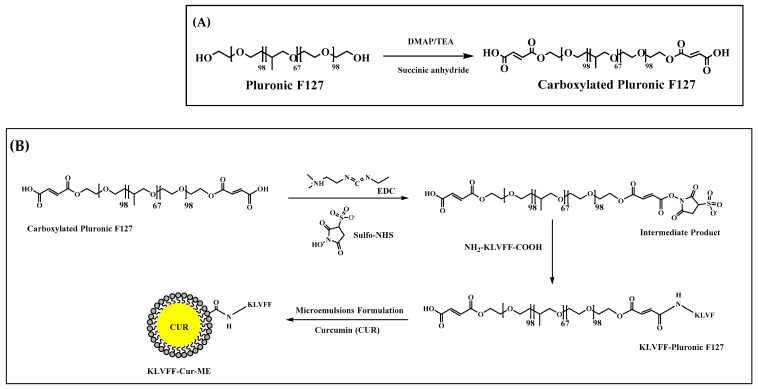
The illustration of synthetic route of carboxylated Pluronic F127 (**A**) Schematic representations of the conjugation reaction of KLVFF peptide with carboxylate Pluronic F127, and formulation of KLVFF-Cur-ME (**B**).

Terminal hydroxyl groups on Pluronic F127^®^ were converted to carboxyl groups according to the following procedure. Pluronic F127^®^ was dissolved in tetrahydrofuran (THF, 60 mL). Then, 4-dimethylaminopyridine (DMAP, 98 mg), triethylamine (108 µL), and succinic anhydride (800 mg) were added. The mixture was stirred for 48 h at room temperature. The solution was dried by rotary evaporation and was dissolved in carbon tetrachloride (30 mL). The excess succinic anhydride was removed by filtration. The COOH-Pluronic F127 was purified by precipitation with ice-cold diethyl ether. The product was identified by FTIR spectroscopy. The illustration of synthetic route of carboxylated Pluronic F127 is shown in Figure 1A.

### 2.3. Synthesis of KLVFF Conjugated COOH-Pluronic F127 (KLVFF-Pluronic F127)

Carboxylated Pluronic was conjugated to the amino groups of KLVFF peptide by a carbodiimide reaction which is mainly used to form amide linkages between amines and carboxylates [40,41]. The Pluronic-COOH was dissolved in deionized water and was allowed to react with 1-ethyl-3-[3-dimethylaminopropyl] carbodiimide hydrochloride (EDC) and sulfo-NHS for 1 h. KLVFF peptide was added to the activated COOH-Pluronic F127 and stirred for 16 h at room temperature. The KLVFF peptide conjugated COOH-Pluronic F127 (KLVFF-Pluronic F-127) was dialyzed against deionized water for 16 h to remove excess of uncoupled peptide, EDC, and sulfo-NHS. The scheme of the conjugation reaction of KLVFF peptide with carboxylate Pluronic F127 is shown in Figure 1B.

### 2.4. Fourier Transform Infrared Spectroscopy (FTIR)

The FTIR spectra were obtained by using a Nicolet iS5 FTIR spectrometer (Thermo Scientific, Waltham, MA, USA), which was operated in the range of 4000–400 cm^−1^. Pluronic and carboxylated Pluronic were physically mixed with potassium bromide and compressed into a disk using a Specac (Kent, UK) hydraulic press before scanning.

### 2.5. Differential Scanning Calorimetry (DSC)

The DSC curves of Pluronic F127, COOH-Pluronic F127, and KLVFF-Pluronic F127 were obtained using a differential scanning calorimeter (DSC8000, PerkinElmer, Waltham, MA, USA) equipped with a heat flow sensor, and joined via the TA Controller TC 15 interface to a computer. Measurements were driven by Pyris™ software version 13.2.1.0007 (PerkinElmer, Waltham, MA, USA). Samples for DSC measurements were weighed using a Mettler Toledo AT 261 (Columbus, OH, USA) microbalance (±0.01 mg) and sealed in 40 µL standard aluminum crucibles with a single hole punched in the lid. The total mass of a sample was between 3 and 5 mg. An empty pan of the same type was employed as a reference. DSC scans of each mixture were performed at a heating rate of 5 °C/min in the temperature range of 25–200 °C. The DSC cell was purged with a stream of nitrogen at a rate of 50 mL/min.

### 2.6. Solubility of Curcumin

To find out the appropriate ratios of oils, surfactants, and co-surfactants as excipients for microemulsions (ME) formulation, the solubility of curcumin in various oils, surfactants, and co-surfactants was investigated by adding excess curcumin into 2 mL of each vehicle. The mixtures were mixed under stirring for 24 h. After equilibrium for 24 h at room temperature, samples were centrifuged at 5000× *g* rpm for 30 min. The supernatant was diluted with ethanol. The concentration of solubilized curcumin was determined spectrophotometrically at the maximum wavelength of 465 nm. Solubility was carried out in triplicate. 

### 2.7. Pseudo-Ternary Phase Diagram Construction

Pseudo-ternary phase diagrams were constructed to determine the region into which the maximum amount of ME formation occurred. The appropriate components from the result of the solubility study were selected to prepare ME by the spontaneous emulsification technique [42]. Surfactants (mixture of TWEEN 80 and KLVFF-Pluronic F127 in the ratio 1:1) and co-surfactants (ethanol, PEG 400, and PG), namely, Smix, were mixed in different ratios to prepare ME according to the area existing in the phase diagram. For the construction of the phase diagram, the mixtures of oil, Smix, and water at different ratios were formulated using a titration method under continuous stirring until a transparent ME was formed. The determination of the ME region was performed by visual observation for the turbidity. The samples were classified as ME when they appeared visually as clear liquids. Pseudo-ternary phase diagrams were drawn using SigmaPlot software version 11.0 (Systat Software, Inc., Chicago, IL USA), the areas of the ME regions were measured by ImageJ 1.47v software (National Institutes of Health, Bethesda, MD, USA).

### 2.8. Curcumin-Loaded KLVFF-Pluronic F127 Microemulsions (KLVFF-Cur-ME)

From pseudo-ternary phase diagrams, the one showing the maximum region for ME was selected to be considered as an optimized ratio for drug-loaded ME formulation. Various formulations were investigated in terms of droplet size, zeta potential, polydispersity index (PDI), transparency, pH, and conductivity to obtain an optimized formulation. The formulation that showed satisfactory results was selected for fabricating curcumin-loaded KLVFF-Pluronic F127 microemulsions (KLVFF-Cur-ME).

KLVFF-Cur-ME was prepared by dissolving curcumin into the oil phase by adding the required quantity of Smix and water and stirring to form a clear and transparent dispersion. The characterizations of KLVFF-Cur-ME were investigated for the percentage of transmittance, globule size, zeta potential, conductivity, and pH.

### 2.9. Physicochemical Characterization of Microemulsions

#### 2.9.1. Conductivity Measurement

The electrical conductivity of ME was measured with a conductivity meter (Metrohm, Switzerland) equipped with a magnetic stirrer. The conductivity measurement was performed using a conductivity cell (with a cell constant of 1.0) consisting of two platinum plates separated by desired distance and liquid between the platinum plates acting as a conductor.

#### 2.9.2. Particle Size and Zeta Potential Measurements

The average droplet size and polydispersity index (PDI) of ME were measured by dynamic light scattering (SZ-100, HORIBA, Kyoto, Japan). All determinations were conducted in triplicate.

#### 2.9.3. Percent Transmittance Measurement

The percent transmittance of the system was checked by measuring transmittance at 650 nm with distilled water as a reference [17,43] by a UV spectrophotometer (UV2600i, Shimadzu, Kyoto, Japan).

#### 2.9.4. pH Measurement

The pH values of ME and COOH-ME were determined using a digital pH meter (pH meter, Metrohm, Herisau, Switzerland), standardized using pH 4 and 7 buffers before use.

### 2.10. Morphology Characterization of KLVFF-Cur-ME

The TEM micrograph of KLVFF-Cur-ME was obtained to characterize morphology (JEM 2010, JEOL, Tokyo, Japan) at an acceleration voltage of 100 kV and 8000× magnification. KLVFF-Cur-ME was stained with a 1% aqueous phosphotungstic acid solution and deposited on the carbon-coated copper grid.

### 2.11. Determination of Curcumin Content in KLVFF-Cur-ME

The curcumin content in KLVFF-Cur-ME was determined using a spectrophotometer at the maximum wavelength of 465 nm. A certain amount of KLVFF-Cur-ME was diluted with ethanol, followed by centrifugation at 5000× *g* rpm for 30 min. The supernatant was taken, and the amount of curcumin was analyzed by a UV–visible spectrophotometer [44].

### 2.12. Ex Vivo Permeation Study

Curcumin permeation study was conducted using Franz diffusion cells (V9-CA, PermeGear, Hellertown, PA, USA) through the porcine nasal mucosa. Freshly excised porcine nasal mucosa, obtained from the slaughterhouse, was immediately soaked in phosphate buffer (pH 6.4) The protocol for the use of cadavers was approved by the Animal Care and Use Committee, Faculty of Veterinary Medicine, Chiang Mai University, Thailand (FVM-CMU-ICUC Ref. No. R5/2563). Fresh porcine nasal mucosa with a thickness of 0.2 mm was mounted between donor and receptor compartments with a volume capacity of 12.5 mL. KLVFF-Cur-ME equivalent to a similar amount of curcumin in the solution was placed into the donor compartment. At the same time, the receptor was filled with phosphate-buffered saline (PBS, pH 6.4), which was maintained at 37 °C under continuous stirring. A one milliliter aliquot was taken at different time intervals and replaced with an equal volume of PBS. After suitable dilution, the sample was analyzed for curcumin content by HPLC.

### 2.13. HPLC Analysis of Permeated Curcumin

The samples obtained from the receptor compartment of Franz diffusion cell were analyzed by HPLC. The separation was performed on a reverse-phase C18 column (250 mm × 4.6 mm, i. d. 5 µm particle size). The elution was carried out with isocratic solvent systems with a flow rate of 1.2 mL/min at ambient temperature. The mobile phase consisted of a 50:50 (*v*:*v*) mixture of acetonitrile and 2% acetic acid in water. The UV detector was set at a wavelength of 424 nm.

### 2.14. Nasal Ciliotoxicity Study

Freshly excised porcine nasal mucosa was obtained from the slaughterhouse and immediately soaked in phosphate buffer (pH 6.4). The cartilage was gently removed to isolate nasal mucosa. Each piece of the porcine nasal mucosa with even thickness (0.2 mm) was mounted on Franz diffusion cell with the positive control (isopropyl alcohol) negative control (PBS pH 6.4) and KLVFF-Cur-ME for 2 h. After that, all pieces of mucosa were rinsed with PBS (pH 6.4) and soaked in a 10% *v*/*v* formalin solution overnight. Each mucosa was 7 mm thick and fixed in paraffin blocks. Fine pieces were stained by eosin and hematoxylin. The prepared slides were observed under an inverted microscope (Motic, AE2000, Richmond, BC, Canada) with a magnification of 10×, captured to evaluate any damage to the nasal mucosa. The protocol for the use of cadavers was approved by the Animal Care and Use Committee, Faculty of Veterinary Medicine, Chiang Mai University, Thailand (FVM-CMU-ICUC Ref. No. R5/2563).

### 2.15. Statistical Analysis

All data were presented as mean ± SEM, *n* = 3 experiments. *t*-test was used to determine a significant difference between the means of the two groups. Statistical analysis of data was completed using an analysis of variance (one-way ANOVA), followed by Newman–Keuls method as a post hoc test to evaluate the significance of differences. In all cases, a value of *p* < 0.05 was considered statistically significant. 

## 3. Results and Discussion

### 3.1. Synthesis and Characterization of COOH-Pluronic F127 and KLVFF-Pluronic F127

**Figure 2:**
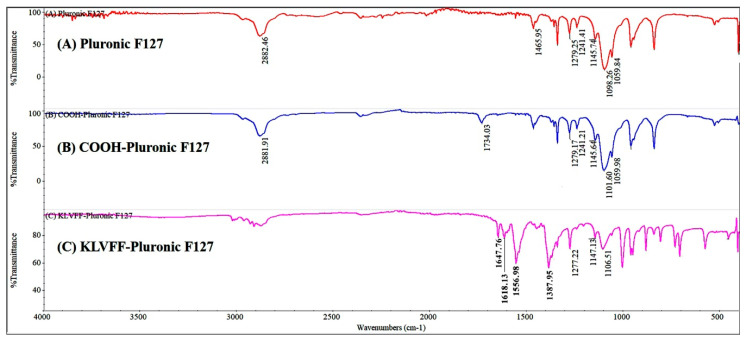
Fourier transform infrared spectra of (**A**) Pluronic F127, (**B**) COOH-Pluronic F127, and (**C**) KLVFF-Pluronic F127.

Carboxylated Pluronic F127 (COOH-Pluronic F127) was derivatized through the reaction of Pluronic-OH with succinic anhydride described in Section 2.2. The percentage yield of COOH-Pluronic F127 was 85.47 ± 0.17%. From the FTIR spectrum (Figure 2), the carbonyl stretch C=O of a carboxylic acid in COOH-Pluronic F127 (blue line) appeared at about 1734 cm^−1^ [45]. In contrast, the carbonyl stretch C=O of a carboxylic acid did not occur on the FTIR spectrum of Pluronic F-127 (red line), indicating the successful transformation of the hydroxyl group of Pluronic F127 to the carboxyl group [46]. Moreover, a strong wideband for the O-H stretch appeared as a broadband in the region 3300–2500 cm^−1^, centered at around 2882 cm^−1^ [47], and the C-O stretch appears at about 1101 cm^−1^ [48] for COOH-Pluronic F127 (blue line) revealed the conversion of OH to COOH. After the KLVFF conjugation (pink line), the typical bands of the amide carbonyl group (O=C-Nh) and imino groups of KLVFF units were observed at 1618 cm^−1^ and 1556 cm^−1^, respectively [49]. The stretching variation absorbance of N-Hat 3200–3479 cm^−1^ [50] and an obvious peak at 1647 cm^−1^ arising from the C=O stretching vibration [50] were observed from the FTIR spectroscopy, supporting the existence of KLVFF.

**Figure 3:**
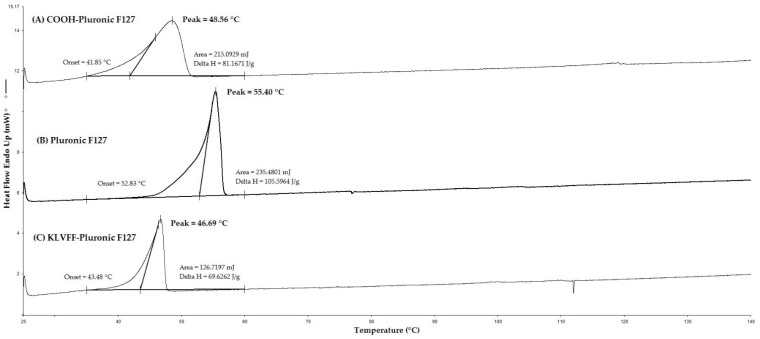
DSC thermogram of (**A**) Pluronic F127, (**B**) COOH-Pluronic F127, and (**C**) KLVFF-Pluronic F127.

Differential scanning calorimetry could be used to determine the phase state of a compound. It can also be used to observe the fusion and crystallization characteristics of polymers [51]. After increasing the temperature, the sample was melted at the melting temperature (Tm) which resulted in an endothermic peak in the DSC curve [51]. The melting endothermic peak that was the temperature at which the solid material melted (Tm) is a very important result for the characterization of any material and especially for the energetic materials [51]. The DSC diagrams are shown in Figure 3; the Tm of Pluronic F127 compared with that of COOH-Pluronic F127 declined from 55.40 to 48.45 °C. The FTIR spectra and DSC thermograms indicated the conversion of OH groups of Pluronic F127 to COOH groups, while the Tm of COOH-Pluronic F127 compared with KLVFF-Pluronic F127 declined from 48.45 to 46.69 °C. The decrease in the Tm of KLVFF.Pluronic F127 might have resulted from the connection of KLVFF to COOH-Pluronic F127.

### 3.2. Solubility of Curcumin

**Figure 4:**
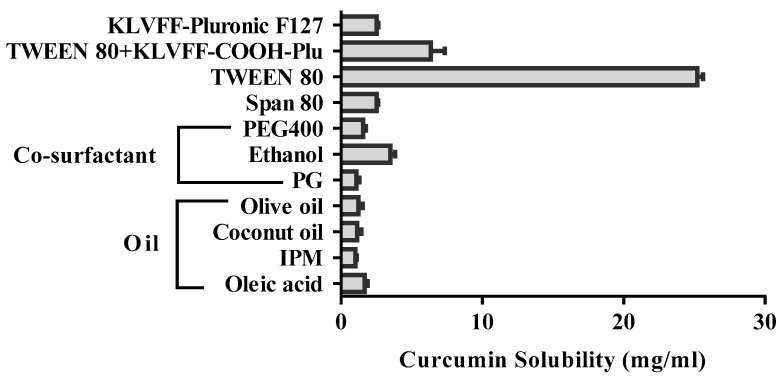
Solubility data for curcumin in oil, surfactant, and co-surfactant.

The solubility of curcumin in various oils and co-surfactants was analyzed to screen the components for microemulsions. Selecting the best oil phase was required for the maximum solubility potential for the drug to cover a larger ME region in the ternary plots and obtain a stable ME [17,52]. Solubility data for the curcumin in oil, surfactant, and co-surfactant are shown in Figure 4. Curcumin showed the highest solubility in oleic acid (1.68 ± 0.21 mg/mL). compared to other oils. Therefore, oleic acid was fixed as the oil phase for further studies. On the other hand, the co-surfactant selected for this study was ethanol, showing the highest solubility (3.50 ± 0.33 mg/mL) for curcumin. TWEEN 80 has been reported to significantly enhance drug concentration in the brain via the intranasal route [53]. Although TWEEN 80 performed the highest solubility (25.20 mg/mL) this study aimed to develop KLVFF-conjugated microemulsions via coupling the amino group of the KLVFF peptide with the carboxylic group on modified Pluronic (Pluronic-COOH). Therefore, the mixture of TWEEN 80 and KLVFF-Pluronic F127 (Tween 80-KLVFF-COOH-Plu) was used as a surfactant in this study, which presented the solubility of curcumin at 6.35 ± 0.94 mg/mL.

### 3.3. Pseudo-Ternary Phase Diagram Construction

**Figure 5:**
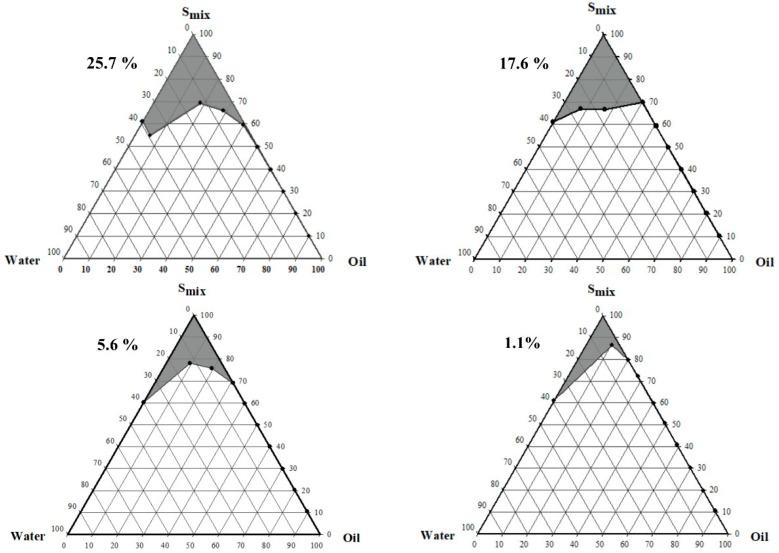
Pseudo-ternary phase diagrams at different ratios of surfactant and co-surfactant (1:1, 2:1, 3:1, 4:1).

The pseudo-ternary phase was constructed from the components that showed the maximum solubility, as shown in Figure 5. The microemulsions system consisted of oleic acid as the oil phase, a mixture of TWEEN 80 and COOH-Pluronic F127 as a surfactant, and ethanol as the co-surfactant. The gray region in the diagram exhibits the microemulsions region. The weight ratios of the surfactant/co-surfactant (Smix) were 1:1, 2:1, 3:1, and 4:1. The largest region (25.7%) was observed in the 1:1 weight ratio of the surfactant/co-surfactant, while the smallest region (1.1%) was presented in the 4:1 weight ratio. The result demonstrated that an increase in the surfactant/co-surfactant mixture ratio resulted in a decrease in the microemulsions region. Our results conformed to the observations reported by other works [54]. Increasing TWEEN 80 promoted the increasing incorporation of water, leading to the turbidity of the system [15]. As described above, the largest microemulsions region was for 1:1 Smix. Therefore, it was selected as the optimal system for incorporating curcumin, revealing formulations F1, F2, F3, F4, and F5, as shown in Table 1.

### 3.4. Physicochemical Characterization of Microemulsions

**Figure 6:**
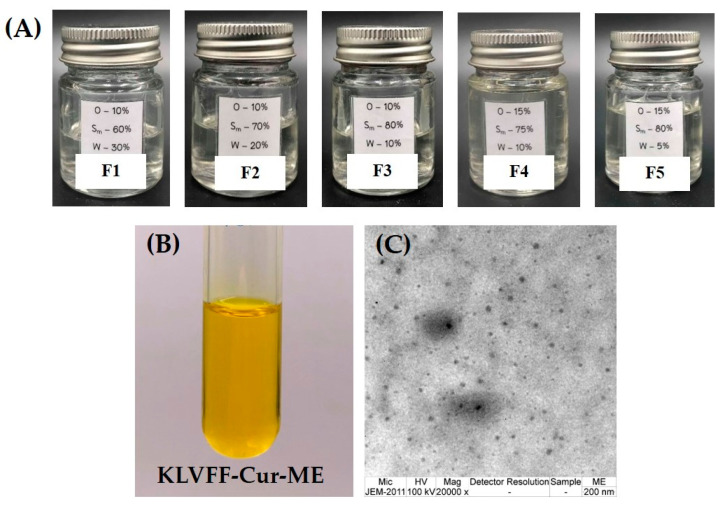
The appearance of blank microemulsions F1–F5 (**A**), KLVFF-Cur-ME (**B**), and a transmission electron microscopy image of KLVFF-Cur-ME (**C**).

Physicochemical and formulation factors are necessary for the rational design of a dosage form [55]. The expected properties of the system for a transnasal administration should have revealed non-irritation, a high permeation, and low clearance from the nasal mucosa. Hence, the physicochemical properties of microemulsions were investigated as tabulated in Table 1 to predict the usability of ME for the transnasal route. The pH value of formulation should have been close to the nasal secretion, reported around 4.5–6.5 [15], suggesting that the formulations would avoid nasal irritation [56]. The viscosity was one of the parameters that should have been considered for a transnasal application, in which low viscosity displays a facile packing, handling, and hassle-free administration of formulations [57]. Although a higher viscosity facilitates the prolonged retention time of formulation at the nasal cavity, the permeation rate decreased with an increased viscosity [15]. Thus, the formulation should have an optimal viscosity. The viscosity of all formulated MEs in this study was around 40–110 cP. Previous reports indicated that a viscosity between 100 and 200 cP is suitable for nasal administration [58]. The percentage transmittance was greater than 95% for all formulated MEs, indicating a clear dispersion and confirmed transparency properties [59]. Moreover, the appearance of all MEs presented a clear and transparent dispersion as shown in Figure 6A, B. These two findings confirmed that all formulated MEs met the criteria of transparency, which was one of the desired properties of microemulsions [13]. Permeation was affected by the globule size, and a faster permeation was expected when the globule size was small [15]. The globule sizes for all formulated MEs were in the range of 55.4–103.0 nm, which is generally considered to be the globule size of a microemulsions (≈10–150 nm) [60]. Nanocarriers with the size range of 10–200 nm were reported to easily transport across the BBB by efficiently encapsulating drug molecules and increasing their diffusion through biological membranes compared to the oral route [14]. MEs have small a droplet size and have a higher surface area compared to other formulations; therefore, they are expected to effectively transport the drug through intranasal delivery [16].

Normally, a PDI of close to zero indicates the uniformity of a globule size, while one indicates a polydisperse sample with multiple size populations [61]. Values of 0.2 and below are most commonly deemed acceptable for polymeric nanoparticles, while 0.3 is considered to be acceptable and indicates a homogenous population of phospholipid vesicles [61]. The results exhibited that PDI values were greater than 0.3, in which the dispersity was likely to be polydisperse. However, the instrument still reported the monodispersity of the samples. The zeta potential values of all MEs were slightly negative (from −0.1 to −0.3 mV). It is generally known that the system is stable when the absolute value of zeta potential is greater than 30 mV, but zeta potential does not fully reflect the stability of the microemulsions [62]. For microemulsions systems containing nonionic surfactants, steric hindrance plays an important role in stability. The stability of microemulsions with a low absolute value of the zeta potential were reported stable [62]. The colloidal stability of MEs with a slightly negative charge was previously discussed [15]. Several reports have shown that MEs containing slightly negative values of the zeta potential have shown a good colloidal stability [17,62,63,64,65]. However, only obtaining the zeta potential value is not enough to predict the stability of formulations, and a stability study should be performed to confirm the stability of the formulation in our future study. Based on the above rationale critical quality attributes, F5 was selected for a further study to formulate KLVFF-Cur-ME. From Table 1, KLVFF-Cur-ME showed a slightly increased internal droplet globule size compared to plain ME (F5). This result indicated that curcumin would immerse in the surfactant film around an oily droplet of o/w microemulsions [60]. This may be attributed to the fact that KLVFF-Cur-ME presented a good quality of microemulsions in terms of the globule size, PDI, pH, viscosity, and % transmittance as described abovw. Moreover, the drug loading content was found to be 80.25 ± 5.47% for KLVFF-Cur-ME, indicating the high encapsulation efficiency of the ME.

### 3.5. Morphology Characterization of KLVFF-Cur-ME

The morphology of KLVFF-Cur-ME using TEM revealed a spherical shape with size in the nanoscale (20–65 nm), as shown in Figure 6C. This result conformed to the globule size distribution measured using photon correlation spectroscopy in Table 1. 

### 3.6. Ex Vivo Permeation Study

**Figure 7:**
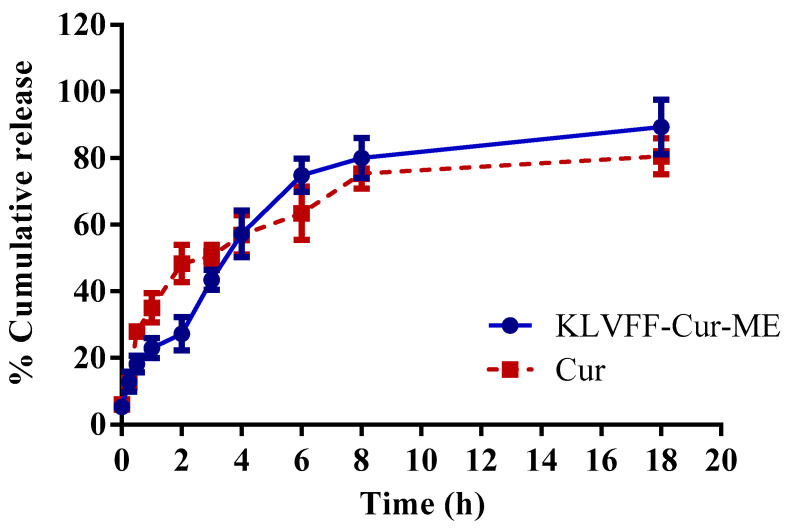
Ex vivo curcumin release profile of curcumin solution and KLVFF-Cur-ME through porcine nasal mucosa membrane.

An ex vivo diffusion study was performed to assess the drug diffusion through a biological membrane simulating the actual in vivo barrier to drug diffusion [66]. The ex vivo drug diffusion study of KLVFF-Cur-ME was studied to acquire a more precise diffusion profile. The ex vivo diffusion profile of free curcumin and KLVFF-Cur-ME is shown in Figure 7, whereby the regression coefficients (*r^2^*) for the zero-order, first-order, and Higuchi models are shown in Table 2. The Higuchi model was better suited to the KLVFF-Cur-ME release rate (*r^2^* = 0.9222), indicating a curcumin partitioning through diffusion, since the porcine mucosa acted as a barrier or controlling membrane [67]. The result indicated that the diffusion process of KLVFF-Cur-ME was closer to the reservoir system than the zero-order (concentration-independent) or first-order (concentration gradient) diffusions [67]. Moreover, the result was also evidenced by the successful diffusion of KLVFF-Cur-ME through the porcine nasal mucosa.

### 3.7. Nasal Ciliotoxicity

**Figure 8:**
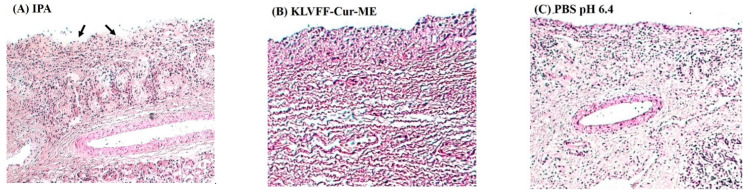
Optical microscopic images (10×) of nasal mucosa treated with: (**A**) positive control—isopropyl alcohol; (**B**) KLVFF-Cur-ME; (**C**) negative control—PBS pH 6.4.

The nasal ciliotoxicity was studied to evaluate the toxic effect of the excipients used for the nasal mucosa formulations. Figure 8A, the porcine nasal mucosa treated with a mucociliary toxic agent (isopropyl alcohol) showed the destruction of the epithelium layer with damage to internal nasal tissues (arrows). Nasal mucosa treated with the negative control (PBS pH 6.4) showed an intact epithelium layer without tissue damage (Figure 8C). KLVFF-Cur-ME did not exhibit toxicity on tissue damage with an intact epithelium layer, indicating the safety of excipients used in the formulation of KLVFF-Cur-ME in this study (Figure 8B). Our results agreed with other reports, demonstrating the intoxicity of oleic acid and TWEEN 80 [15].

## 4. Conclusions

In this study, Cur-ME was prepared using the titration method. A carbodiimide reaction was performed for the KLVFF conjugation. KLVFF-Cur-ME was successfully synthesized with acceptable physicochemical characteristics and was suitable for intranasal administration. The encapsulation of curcumin into the microemulsions improved the solubility and released the profile of curcumin. KLVFF-Cur-ME did not show nasal ciliotoxicity. The kinetic release profile of KLVFF-Cur-ME followed the Higuchi model. The result from the ex vivo permeation study suggested the successful diffusion of KLVFF-Cur-ME through the porcine nasal mucosa. Therefore, KLVFF-Cur-ME may provide a new approach for inhibiting A*β* aggregation via intranasal administration. However, the potential of the developed KLVFF-Cur-ME for nose-to-brain delivery of curcumin can only be established after in vivo toxicity and biodistribution studies.

## Figures and Tables

**Table 1:** Physicochemical properties of ME.

Formulation	Oil: Smix: Water	Globule size (nm)	PDI	Zeta Potential (mV)	Viscosity (cP)	%T	pH	Conductivity (ms/cm)
F1	10:60:30	70.8 ± 0.9	0.333 ± 0.037	−0.02 ± 0.01	72.89 ± 2.04	95.64 ± 0.55	4.85 ± 0.03	0.02 ± 0.00
F2	10:70:20	80.7 ± 3.8	0.494 ± 0.017	−0.1 ± 0.1	53.44 ± 0.69	95.81 ± 0.12	4.96 ± 0.01	0.02 ± 0.06
F3	10:80:10	55.4 ± 3.3	0.504 ± 0.017	−0.1 ± 0.1	49.78 ± 3.34	96.06 ± 0.43	4.88 ± 0.05	0.02 ± 0.00
F4	15:75:10	103.0 ± 2.9	0.316 ± 0.003	−0.3 ± 0.3	50.44 ± 2.04	95.43 ± 0.43	5.16 ± 0.04	0.02 ± 0.01
F5	15:80:5	64.6 ± 2.7	0.471 ± 0.014	−0.1 ± 0.2	78.46 ± 5.58	95.39 ± 1.63	4.90 ±0.03	0.02 ± 0.00
KLVFF-Cur-ME	15:80:5	76.1 ± 2.5	0.405 ± 0.015	−0.1 ± 0.7	70.98 ± 3.02	97.03 ± 0.01	5.33 ± 0.02	0.02 ± 0.01

**Table 2:** Release kinetics of curcumin and KLVFF-Cur-ME.

	*r^2^*		
Formulation	Zero-Order	First-Order	Higuchi
Curcumin	0.6621	0.4100	0.9044
KLVFF-Cur-ME	0.7494	0.5434	0.9222

## Data Availability

Not applicable.
